# Clinical and neurophysiological risk factors of learning disabilities in different types of idiopathic focal epilepsy

**DOI:** 10.22037/ijcn.v15i4.32071

**Published:** 2022-03-14

**Authors:** Al Amir BASSIOUNY MOHAMED, Gharib FAWI, Yasser WASSEL, Sania ABDELHAMEED, Ahmed MOUSA, Ghada HUSSEIN, Ahmed BORAI

**Affiliations:** 1 Department of Neurology and Psychological Medicine, Faculty of Medicine, Sohag University Hospital, Sohag, Egypt; 2 Neurology Department, Sohag Faculty of Medicine, Sohag University, Sohag, Egyp; 3 Department of Surgery, College of Medicine, King Khalid University, Abha, and from the Neurology Unit (Alhazzani), Department of Medicine, College of Medicine, King Saud university, Riyadh, Kingdom of Saudi Arabia; 4 Department of Ophthalmology, College of Medicine, King Saud University, Riyadh, Saudi Arabia; 5 Faculty of Science, Suez University, Suez, Egypt

**Keywords:** Stroke, learning disabilities, academic underachievement, epileptic focus

## Abstract

**Objectives:**

Academic difficulties are common in epileptic children. A learning disability (LD) is a reduction in the learning capacity of children or the intellectual ability of adults, which is different from mental retardation or dementia.

**Materials & Methods:**

The participants were 56 patients, of whom 35 were males (62.5%), and 21 were females (37.5%). The participants attended the Neurology Outpatient Clinic, the Sohag University Hospital, between December 2016 and May 2017. Children with chronological age between 7 and 16 years with idiopathic focal and normal mental and motor developmental history were enrolled in this cross-sectional study. The Revised Quick Neurological Screening Test was used to assess different types of LD.

**Results:**

LDs were present in 67.9% of our participants with a statistically significant association between LDs in one arm and younger age, earlier age of onset of epilepsy, frequent seizures, and seizure semiology, particularly of temporal lobe origin, in the other arm. In addition, left epileptic focus on EEG, prolonged treatment duration with antiepileptic drugs (AED), and polytherapy were significantly correlated with LDs.

**Conclusion:**

Many factors are significantly correlated with LDs in children with idiopathic focal epilepsy, like age of the patient, age of epilepsy onset, seizure semiology, prolonged AED treatment, and polytherapy.

## Introduction

As a common neurological condition, epilepsy can occur at any age, with a point prevalence of active epilepsy being 6.38 per 1,000 persons (1). For the diagnosis of epilepsy, at least two unprovoked seizures are required 24 hours apart, or one unprovoked with a high risk of recurrence **(**2**, **3**)**. Focal epilepsy is classified according to the presence or absence of awareness, associated motor behavior (motor and non-motor), and focal to bilateral tonic-clonic seizures **(**4**, **5**)**. A learning disability is a type of neurodevelopmental disorder that impedes the capacity to learn or use specific academic skills (e.g., reading, writing, or arithmetic) during the early years of education **(**6**). **Academic difficulties, particularly in arithmetic, spelling, and reading, are common in epileptic children **(**7**, **8**)**. 

It was also documented that learning problems were more prevalent in children with focal epilepsy than in those with generalized epilepsy and in symptomatic syndromes compared with idiopathic or cryptogenic ones **(**9**-**12**)**.

The Quick Neurological Screening Test (QNST) is a neuropsychological test for screening learning disorders** (**13**)**. The majority of the previous studies use IQ as an indicator of learning disabilities, and most of them enroll patients with both focal epilepsies (including idiopathic and symptomatic) and generalized epilepsies. This cross-sectional study aimed to investigate the effect of clinical and neurophysiological risk factors of LDs on idiopathic focal epilepsy.

## Materials & Methods

Participants were recruited from the Neurology Outpatient Clinic, the Sohag University Hospital, Egypt, between December 2016 and May 2017. The sample (n=56, 35 males (62.5%) and 21 females (37.5%)) consisted of children with a diagnosis of idiopathic focal epilepsy with a chronological age between 7 and 16 years and an intelligence quotient (IQ) above 85 (normal mentality)** (**11**)**. 

Children with mental and motor neurological disorders were excluded from the study, and those on phenytoin or topiramate were also ruled out because of possible cognitive side effects. Each patient was subjected to full medical and neurological evaluation. The educational stages were classified according to the International Standard Classification of Education to primary and secondary education (Table 1). 

Focal motor epilepsy has been classified as focal awareness, focal with impaired awareness, and focal to bilateral tonic-clonic epilepsies **(**14**)**. 

Seizure frequency has been classified according to the number of seizures per year to 0-1, 2-9, and ≥ 10/year **(**15**)**.

Uncontrolled seizures are defined as more than two changes in AED therapy and then ≥1 epilepsy-related emergency department (ED) visit/hospitalization within one year, while controlled seizures are defined as no AED change and no epilepsy-related ED visit/hospitalization **(**15**, **16**)**.

The study was approved by the Ethical Committee at the University of Sohag in March 2015, and informed written consent was taken from each patient or one of their relatives. 

We followed the methods of Gharib Fawi et al. (2019) **(**17**)**, who used the Revised Quick Neurological Screening Test (QNST-R) as a screening tool for LDs. QNST-R consists of 15 subtests ranging from 0 to 149 (Table 2). A total score of 25 or less is considered normal, but the score exceeding 50 is considered high)** (**13**).** Mental retardation was excluded in the participant children using the validated Arabic version of the Wechsler Intelligence Scale for Children fourth edition (WISC-IV) **(**17**)**. Electroencephalography (EEG) was obtained by using a 10~20 system with a minimum duration of 20-30 minutes and looking for background activity and site of epileptic focus **(**18**)**

**Statistical analysis**

Statistical Package for the Social Sciences for Windows (SPSS 20.0, IBM Corp., Armonk, NY, USA) was used. Descriptive statistics were used to investigate the general characteristics of the epileptic patients. Continuous data were expressed as mean ± SD, and categorical data were expressed as numbers and percentages. Student's t-test was used for the continuous data, and Chi-square test for categorical data was used to detect different clinical and neurophysiological risk factors for LDs. The relationship between LDs and different patient variables was investigated using Spearman’s correlation coefficient, and preliminary analyses were performed to ensure no violation of normality, linearity, and homoscedasticity assumptions. P values of less than 0.05 were considered significant.

## Results

The study included 56 patients (35 males (62.5%) and 21 females (37.5%)) with idiopathic focal epilepsy. LDs were present in 67.9% of our participants, and different characteristics of patients and controls are presented in Table (3). The mean age of epileptic patients with and without LD was 11.32±2.7 versus 13±2.8, respectively (P-value = 0.033). LDs were found in 63.2% of the males and 36.8% of the females and predominantly in 4-6 academic years, as shown in Table (4).

LDs were significantly related to younger age, earlier age of epilepsy onset, more frequent seizures, focal epilepsy, left epileptic focus on EEG, longer treatment duration, and polytherapy (Table 4,5).

## Discussion

Epilepsy is an important health problem affecting more than 50 million people worldwide **(**22**, **23**)**. School underachievement is more common in children with epilepsy **(**19**)**, and more than 60% of patients suffer from school-related difficulties **(**20**)**. To the best of our knowledge, the previous studies that are directed to elucidate the risk factors of LDs in idiopathic focal epileptic patients without mental retardation are lacking. 

In this work, we found that about sixty-seven percent of our patients had LDs (as measured with QNST-R), which is confirmed by Colin Reilly et al**. (**7**), **who documented that seventy-two percent of children with epilepsy displayed low academic achievement. In addition, Sillanpää et al. reported LDs in 57% of patients with an IQ greater than 85 **(**11**)**. Finally, Fastenau et al. **(**21**)** observed that 48.2% of children with epilepsy met the psychometric criteria for LDs. In contrast, Deonna et al. **(**22**) **reported that only 40.9% of epileptic children had school difficulties after controlling the effect of antiepileptic drugs, and this difference may be attributed to the use of different methods to assess LDs. 

This study demonstrated that the younger age of children was significantly associated with LDs, and this result is similar to that obtained from several studies **(**12**, **23**, **24**)**. However, this finding is not similar to that obtained in other studies ** (**19**, **20**)** in which children with generalized and focal epilepsies were enrolled and different tests, namely the Bender test and the teacher report, were used to assess school achievements. 

The current study demonstrated that earlier age of epilepsy onset was significantly associated with LDs, which is in accordance with the results obtained by Schoenfeld et al**. (**25**)**, who affirmed that a patient's age was strongly correlated with the academic performance in children with epilepsy. In addition, Zelnik et al. **(**9**)** documented that young age at onset was among the predictors for special education for epileptic kids. Also, Huttenlocher et al. **(**26**)** revealed that cognitive impairment was worse in epilepsy during early development than in the mature brain. This finding is clarified in some animal studies reporting that early seizures may profoundly impact the development of the maturing brain** (**27**-**29**)**. Furthermore, the immature brain is more inclined to seizures than is the mature brain because of an imbalance between excitation and inhibition mechanisms and various physiologic and structural features** (**30**, **31**)**. Other studies failed to find a significant relationship between LDs and young age at the onset because of different sample sizes and the enrollment of all children with epilepsy, and not an idiopathic focal one, as we recruited **(**19**, **20**, **32**-**34**)**. 

We found that LDs were significantly correlated with frequent seizures, and this finding is confirmed by previous studies reporting that poor seizure control appears to be associated with decreased academic performance, particularly reading achievement and attention problems **(**9**, **12**, **34**)**. Other studies have failed to show strong relationships between seizure frequency and academic achievement **(**20**, **25**, **32**, **33**, **35**)**, and this is because they used different psychological methods, namely word span, for learning evaluation. We found that left epileptic focus was significantly associated with LDs, which is similar to the results obtained in previous studies **(**10**, **36**, **37**)**. Similarly, another study reported that the seizure onset in the language-dominant hemisphere, as compared with the nondominant hemisphere, was associated with higher rates of specific learning disabilities** (**38**)**. In contrast to other studies **(**25**, **39 that failed to find a significant association between LDs and hemisphere of seizure focus** (**25**, **39**)**, these studies' participants were confined to those with temporal lobe epilepsy only. 

This work confirmed that a longer duration of epilepsy is significantly associated with LDs, which agrees with previous studies **(**12**, **24**)**. Also, we demonstrated that polytherapy was significantly correlated with LDs, which is similar to Cornaggia and Gobbi, who reported that antiepileptic drugs might cause state-dependent (potentially treatable and reversible) learning disorders **(**40**)** and Al-Twajri et al. **(**41**)**, who observed higher rates of LDs in epileptic children on polytherapy compared to monotherapy. The mechanisms by which epilepsy leads to LDs are probably the direct effects of seizures on the maturing brain of children, the effects of concomitant neuropsychological deficits, and finally, the adverse effects of drug therapy **(**11**)**. This study has some restrictions that should be taken into account in evaluating LDs in epileptic children. For instance, we did not follow up patients to clarify the role of antiepileptic drugs in controlling seizure activity or education programs for LDs. Also, the specifications of LDs were unknown. Despite these limitations, the present study highlighted the importance of LDs in a particular sector of epilepsy, which should be considered when dealing with children with epilepsy to minimize academic underachievement. Still, these results need to be confirmed in a more extensive and detailed study.

**Table (1):** The International Standard Classification of Education (ISCED) ISCED

**Year**	**Age**	**School**	
Kindergarten	4–5	Preschool	
Prep	5–6		
Grade/Year 1	6–7	Lower Primary	Primary
Grade/Year 2	7–8		
Grade/Year 3	8–9		
Grade/Year 4	9–10	Upper Primary	
Grade/Year 5	10–11		
Grade/Year 6	11–12		
Grade/Year 7	12–13	Junior Secondary	Secondary
Grade/Year 8	13–14		
Grade/Year 9	14–15		
Grade/Year 10	15–16	Senior Secondary	
Grade/Year 11	16–17		
Grade/Year 12	17–18		

**Table (2):** Subtests of the Revised Quick Screening Test (QNST-R)

**Subtest**	**Description**
**l. Hand skill **	The subject is instructed to write his or her name and an age-appropriate six- to eight-word simple sentence.
**2. Figure recognition ** **and production **	Present page containing a series of five geometric figures on the recording form. The subject is instructed to name each one and then draw them.
**3. Palm form recognition **	Subject is instructed to identify, solely by touch, numerals drawn on the palm of his or her hands.
**4. Eye tracking **	Present a pencil or other appropriate object at Subject 's eye level. The subject is instructed to follow it back and forth.
**5. Sound patterns **	The subject is instructed to reproduce sound patterns with the eyes closed manually and orally after the patterns are demonstrated by the examiner.
**6. Finger to nose **	Subject is instructed to close both eyes and reach back and forth between examiner's hand and the tip of his or her own nose.
**7. Thumb and finger circle **	The subject is instructed to form successive circles by touching the thumb to each of the fingers.
**8. Double simultaneous stimulation of hand and cheek **	Examiner observes whether subject is able to feel the gentle simultaneous touch on both hands, bilateral cheeks, and one hand and the contralateral cheek.
**9. Rapidly reversing repetitive hand movements **	The subject is instructed to turn his or her hands over rapidly and repetitively after these movements are demonstrated by the examiner.
**10. Arm and leg extension **	The subject is instructed to extend his or her extremities in front of him or her as straight as possible in sitting position.
**I l. Tandem walk **	The subject is instructed to walk a straight line for at least 10 feet, placing the heel of each shoe directly against the toe of the opposite foot. Subject then walk backward on the "line", heel-to-toe, and then repeats the tandem walk forward with his or her eyes closed.
**12. Stand on one leg **	The subject is instructed to balance himself or herself with the eyes open and closed on each foot for a count of 10 seconds.
**13. Skip **	Subject is instructed to skip across the room.
**14. Left-right discrimination **	This section is scored from parts of three other subtests (6, 7, 12). The left-right discrimination is determined by observing whether subjects hold up the right hand (leg) when E uses the right hand (leg) to demonstrate.
**15. Behavioral irregularities **	The final item requires general observation of subject's behavior (E.g., excessive talking, fidgeting, distractibility, defensiveness, anxiety, etc.) during the entire test.

**Table (3):** General characteristics of participants

	**Mean **	**Number and percentage of patients**
**Age** (year)	11.8 ± 2.8	-
**Sex**		
Male	-	35 (62.5%)
Female	-	21(37.5%)
**Seizure frequency**		
0-1 seizure/year	-	24 (42.9)
2-9/year	-	8 (14.3)
>=10/year	-	24 (42.9)
**Educational Years**	-	
1- 3 years	-	13(23.2%)
4-6 years	-	19(33.9%)
7-9 years	-	9(16.1%)
10-12 years	-	15(26.8%)
**Seizure duration**	-	()
1-5 Years	-	34 (60.7)
6-10 years	-	12 (21.4)
>10 years	-	10 (17.9)
**Focal epilepsy subtypes**	-	()
Focal aware	-	29 (51.8)
Focal with impaired awareness	-	27 (48.2)
**EEG focus**		
Left Focus	-	31 (55.4)
Right Focus	-	25 (44.6)
**Antiepileptic drugs**		
Levitracetam	-	16 (28.6)
Carbamazepine	-	22 (39.3)
Valproate	-	18 (32.1)
**Treatment Duration**		
1-5 Years	-	21 (37.5)
6-10 years	-	20 (35.7)
>10 years	-	15 (26.8)
**Number of antiepileptic drugs**		()
Monotherapy	-	40 (71.4)
Polytherapy	-	16 (28.6)
**QNST-R**		
Mean ± SD	58.9±22.2	
Normal	-	18(32.1%)
Abnormal (Learning Difficulty)	-	38(67.9%)

**Table (4):** different patient characteristics in epileptic patients and learning disabilities

	**Normal QNST-R ** **(N=18)**	**Abnormal QNST-R (N=38)**	**P-value**
**Age (year) **	13 ±2.8	11.32±2.7	0.033
**Sex**			0.88
Male	11 (61.1%)	24 (63.2%)	
Female	7 (38.9%)	14 (36.8%)	
**Educational Years**			0.088
1- 3 years	3 (16.7%)	10 (26.3%)	
4-6 years	3 (16.7%)	16 (42.1%)	
7-9 years	4 (22.2%)	5 (13.2%)	
10-12 years	8 (44.4%)	7 (18.4%)	
**Age of Onset**			0.002
<2 years	1 (5.6%)	11 (28.9%)	
2-10 years	7 (38.9%)	22 (57.9%)	
10-16 years	10 (55.6%)	5 (13.2%)	
**Seizure duration **			0.985
1-5 (Years)	11 (61.1%)	23 (60.5%)	
6-10 (Years)	4 (22.2%)	8 (21.1%)	
>10 (Years)	3 (16.7%)	7 (18.4%)	
**Seizure Frequency**			
0-1/year	12 (66.7%)	12 (31.6%)	
2-9/year	3 (16.7%)	5 (13.2%)	
≥10/year	3 (16.7%)	21 (55.3%)	0.020
**Focal epilepsy subtypes **			0.035
Focal aware	13 (72.2%)	16 (42.1%)	
Focal with impaired awareness	5 (27.8%)	22 (57.9%)	
**EEG focus **			0.03
Left Focus	5 (27.7%)	26 (68.4%)	
Right Focus	12 (72.3%)	13 (31.6%)	
**Antiepileptic drugs **			0.041
Levitracetam	9 (50.0%)	7 (18.4%)	
Carbamazepine	4 (22.2%)	18 (47.4%)	
Valproate	5 (27.8%)	13 (34.2%)	
**Treatment Duration**			0.015
1-5 Years	11 (61.1%)	10 (26.3%)	
6-10 years	6 (33.3%)	14 (36.8%)	
>10 years	1 (5.6%)	14 (36.8%)	
**Number of AED**			0.047
Monotherapy	16 (88.9%)	24 (63.2%)	
Polytherapy	2 (11.1%)	14 (36.8%)	
**AED= Antiepileptic drugs; EEG** electroencephalogram; FL=Frontal Lobe;QNST-R =Revised Quick neurological screening test**;**TL = temporal lobe			

**Table (5):** Spearman's correlation between LDs and patient’s variables

	**Correlation Coefficient**	**P value**
age	-0.294	0.028
Educational years	-0.288	0.032
Age of onset of epilepsy	-0.447	0.001
Seizure duration	-0.030	0.827
Seizure frequency	0.372	0.005
Seizure Semiology	0.281	0.036
Origin of epileptic focus	0.229	0.090
Epileptic focus on EEG	-0.308	0.021
AED duration	0.388	0.003
Number of AED	0.266	0.048

## Authors' contribution

All authors read and approved the final manuscript.

## Conflict of interest

There were no financial or non-financial conflict of interest.
